# Immune Dysfunction and Multiple Treatment Modalities for the SARS-CoV-2 Pandemic: Races of Uncontrolled Running Sweat?

**DOI:** 10.3390/biology9090243

**Published:** 2020-08-24

**Authors:** Ashish Kothari, Vanya Singh, Uttam Kumar Nath, Sandeep Kumar, Vineeta Rai, Karanvir Kaushal, Balram Ji Omar, Atul Pandey, Neeraj Jain

**Affiliations:** 1 Department of Microbiology, All India Institute of Medical Sciences, Rishikesh 249203, India; ashishkothari1212@gmail.com (A.K.); singh.vanya26@gmail.com (V.S.); 2 Department of Medical Oncology & Hematology, All India Institute of Medical Sciences, Rishikesh 249203, India; nath.uttam@gmail.com; 3 School of Medicine, Tulane University, New Orleans, LA 70112, USA; sschaudhary55@gmail.com; 4 Department of Entomology & Plant Pathology, North Carolina State University, Raleigh, NC 27695, USA; vrai@ncsu.edu; 5 Department of Biochemistry, All India Institute of Medical Sciences, Rishikesh 249203, India; karanvirkaushal@gmail.com; 6 Department of Ecology, Evolution and Behavior, The Alexander Silberman Institute of Life Sciences, The Hebrew University of Jerusalem, Jerusalem 91904, Israel

**Keywords:** COVID-19, adoptive cell-based therapy, immunomodulatory drugs, cytokine storm, extracellular vesicles, anti-neoplastic regimen, mesenchymal stem cells

## Abstract

Severe acute respiratory syndrome coronavirus 2 (SARS-CoV-2) caused a global pandemic threat with more than 11.8 million confirmed cases and more than 0.5 million deaths as of 3 July 2020. Given the lack of definitive pharmaceutical interventions against SARS-CoV-2, multiple therapeutic strategies and personal protective applications are being used to reduce the risk of high mortality and community spread of this infection. Currently, more than a hundred vaccines and/or alternative therapeutic regimens are in clinical trials, and some of them have shown promising results in improving the immune cell environment and controlling the infection. In this review, we discussed high-performance multi-directory strategies describing the uncontrolled deregulation of the host immune landscape associated with coronavirus disease (COVID-19) and treatment strategies using an anti-neoplastic regimen. We also followed selected current treatment plans and the most important on-going clinical trials and their respective outcomes for blocking SARS-CoV-2 pathogenesis through regenerative medicine, such as stem cell therapy, chimeric antigen receptors, natural killer (NK) cells, extracellular vesicular-based therapy, and others including immunomodulatory regimens, anti-neoplastic therapy, and current clinical vaccine therapy.

## 1. Introduction

SARS CoV-2 is the most recent (2019–2020) and third pandemic zoonotic coronavirus infection (COVID-19). In the past two decades, there have been two other episodes of pathogenic coronavirus infections reported, namely SARS-CoV (2002–2003) and MERS-CoV (Middle East respiratory syndrome coronavirus) (2012), which led to globally high morbidity and mortality [1]. Current management of SARS-CoV-2 infection directed by the government is social distancing, the shut-down of non-essential services, quarantine, large-scale viral testing, fueling researchers to develop new therapeutic interventions, and supportive treatment to prevent further community spread of this virus, which otherwise could have caused uncontrollable large-scale mortality [2]. The severity of SARS-CoV-2 infection ranges from asymptomatic (near to 60%) to mild symptoms of the characteristically disturbed respiratory tract along with fever, cough, and shortness of breath, while others develop severe acute respiratory distress syndrome (ARDS) in almost 5–15% of patients [3]. Activation of cytokine storm has been clinically detected in severe SARS-CoV-2-infected patients—which plays a critical role in the process of disease aggravation through deregulated immune cell function and number—and is considered to be one of the major causes of ARDS and/or multi-organ failure [4,5]. Therefore, effective blocking of cytokine storm can prevent the deterioration of SARS-CoV-2-infected patients. In this scenario, providing care to the immunocompromised patient—especially to those suffering from cancer—is very challenging, as these are in an advanced position to becoming infected by SARS CoV-2, as compared to the general population, so this requires the planned organization of the healthcare system at an unprecedented scale [6]. World-wide high-throughput on-going research and several clinical trials are operational in the direction of identifying the cure of this deadly pathogen. Still, we are in hiatus for an effector regimen. In this review, we have focused our discussion on immunological response and its association with anticancer drugs in the context of SARS-CoV-2 infection. We have elaborated our argument towards most important on-going clinical trials on cell-based therapy: mesenchymal stem cells (MSCs), NK cells, chimeric antigen receptor (CAR)-based; immunomodulatory cytokines, anti-neoplastic regimens, DNA/RNA-based vaccines, and preventive care for the treatment for SARS-CoV-2 infection.

## 2. Immune Response in SARS-CoV-2 Pathogenesis

Lung alveolar epithelial cells are considered as primary entry targets cells for the SARS-COV-2 through the angiotensin-converting enzyme-2 (ACE2) receptor, though it is expressed by numerous tissues [7]. While limited data are available on how SARS-COV-2 escapes the host immune response, by considering the aforementioned SARS-CoV and MERS-CoV, we may extrapolate this knowledge, as SARS-COV-2 shares nearly 80% of its genome with SARS-CoV and has high homology in amino acid sequences for almost all encoded proteins [8]. Both SARS-COV-2 and SARS-CoV utilize the ACE2 receptor to begin infection despite amino acid variation at a specific residue in the ACE2 receptor, suggesting that these variations might have been selected or could have increased the virulence and transmissibility of SARS-COV-2 compared to SARS-CoV [9,10].

### 2.1. Innate and Adaptive Immune Cells in COVID-19 Patients

SARS-CoV-2 infection activates both innate and adaptive immune response, where severe inflammatory response may cause local tissue damage (acute lung injury) and ARDS at systemic level [11,12]. Therefore, the knowledge behind this enhanced activation of cytokine storm due to dysregulated immune function after SARS-COV-2 infection will provide ways to clinically manage and prevent its transmission from mild to severe stage. Notably, bronchial mucosal-associated invariant T (MAIT) cells and γδ T cells are the primary innate immune cells that can trigger cytokines response after SARS-COV-2 infection, especially in patients developing the severe disease [13]. As a result of the activation of these innate immune cells and the consequential expression of pro-inflammatory cytokines genes, the host adaptive immune system becomes activated against virus infection.

Circulating white blood cells, including neutrophil, basophils, eosinophils numbers are consistently higher in survivors of COVID-19 than in non-survivors [14,15]. SARS-CoV-2 infection also induces lymphocytopenia in clinically severe patients that mostly affects the CD4+, CD8+ T cell subset, including effector, memory, regulatory T cells, and natural killer cells [11,12]. These observations are in line with the previous findings in severe or lethal cases of SARS-CoV and MERS [16,17]. This reduction in immune cells repertoire could be due to damage of lymphocytes or lymphocytic organs associated with infection with SARS-CoV-2 via their minimally expressed ACE2 receptors. Notably, the baseline levels of CD8+ T cells and NK cells are inversely correlated to ACE2 expression in human lung tissue [18]. Cytotoxic T lymphocytes (CTLs) and natural killer (NK) cells are considered as a critical cell that controls viral infection. These cells were found to be exhausted in SARS-CoV-2-infected patients with a significant increase in the exhaustion marker PD1 (programmed death-1) compared to healthy controls and thus likely responsible for viral progression [19,20,21]. A recent study from Yang et al. demonstrated clinical outcomes of 93 SARS-CoV-2-positive patients in association with neutrophil(NEU)-to-lymphocyte (LYM) ratio (NLR), lymphocyte-to-monocyte (MON) ratio, platelet-to-lymphocyte ratio (PLR), and C-reactive protein (CRP) expression. According to their study, the aged patient group with elevated NLR was significantly associated with illness severity and could represent an independent prognostic biomarker for COVID-19 patients [22]. Similarly, another group in China with a cohort of 245 SARS-CoV-2-positive patients identified a patient group with elevated NLR, which was at a hyper-risk side compared to the other groups [23].

### 2.2. High-Throughput Sequencing Approach to Understand Immune Cell Dysfunctions in SARS-CoV-2-Infected Patients

Recently, Wen et al. have comprehensively characterized changes in transcriptional landscape during the recovery stage of SARS-CoV-2 infection by single-cell RNA sequencing (scRNA-seq) using peripheral blood mononuclear cells (PBMC) [24]. According to their study, inflammatory cytokines gene expressing CD14+ monocytes and plasma B-cell numbers were remarkably increased in COVID-19 patients during the early recovery stage [24]. Mingfeng et al. characterized bronchoalveolar lavage fluid (BALF)-immune cells through scRNA-seq from patients with varying severity of SARS-CoV-2 (mild and severe). BALF from critical/acute SARS-CoV-2-infected patients showed a higher proportion of macrophages and neutrophils and a lower proportion of myeloid/plasmacytoid dendritic cells and T cells compared with moderately infected patients [25]. Another study using scRNA-seq data identified specific cell types expressing a receptor for coronavirus infection (ACE2) across 13 tissue types; these include lung alveolar cells type-2, liver cholangiocyte, colon colonocytes, esophagus keratinocytes, stomach epithelial cells, and kidney proximal tubules [26]. Importantly, it has been observed that disease conditions, such as chronic heart disease or chronic cigarette smoke exposure, showed enhanced ACE2 expression notified by scRNA-seq data in cardiomyocytes or human lung cells, respectively [27,28]. Notably, human testicular cells (spermatogonia, Leydig, and Sertoli cells) also predominantly express the ACE2 receptor [29] representing that these tissue-specific cells are vulnerable to SARS-COV-2 infection [30]. It is important to note that not only over- or under-expression of ACE2 receptor in human tissue could determine the susceptibility of patient cells to SARS-COV-2, but also ACE2 polymorphism could influence both the predisposition to infection and clinical outcome of the COVID-19 pathogenesis [31]. Using mass-spectrometry, one study has profiled cellular immune components from SARS-CoV-2-infected patients with differences in disease progression (mild, severe, and critical) and compared with peripheral blood cells collected from healthy donors. According to their study, CD8+ T cells, dendritic cells, and macrophages were excessively activated initially during mild disease. They became exhausted in later critical stages, thereby representing disturbed homeostasis of the immune system during disease symptoms progression [32].

The scRNA-seq approach has also been utilized to identify novel therapeutic regimens for SARS-CoV-2 infection. In this line, a bioinformatics pipeline has been developed, which integrates the scRNA-seq dataset with a drug perturbation database to identify potential therapeutic candidates for SARS-CoV-2 infection treatment. Using bioinformatics pipeline, four drugs—(1) didanosine, an HIV anti-viral drug; (2) benzyl-quinazoline-4-yl-amine, an EGFR inhibitor; (3) camptothecin, a topoisomerase inhibitor; and (4) RO-90-7501, an amyloid-β42 aggregation inhibitor—have been proposed as potential candidates to treat COVID-19 [33]. Using knowledge from scRNA-seq and B-cell VDJ sequencing data, Cao et al. developed a therapeutic SARS-COV-2 neutralizing antibody (BD-368-2), with strong therapeutic and prophylactic efficacy in SARS-COV-2, and infected hACE2-transgenic mice [34]. Further, using cryoelectron microscopy, the structure of BD-368-2 in complex with spike-ectodomain trimer was characterized, which revealed BD-368-2 binding to the ACE2 receptor [34].

### 2.3. Hyper-Cytokine Activation

**Figure 1:**
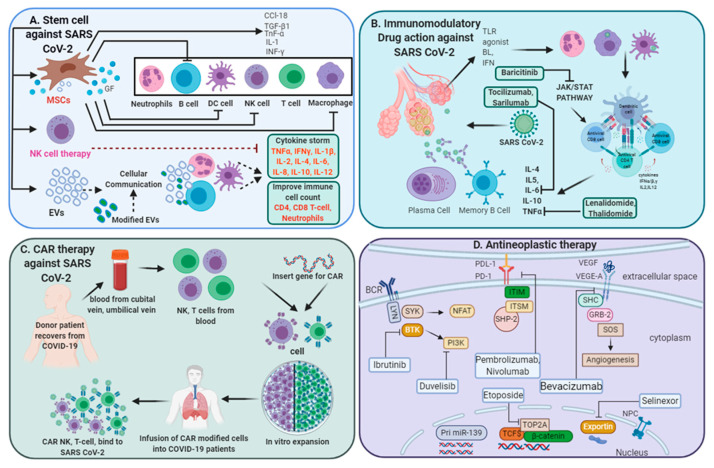
Multi-directive therapeutic intervention currently in clinical trials for combating SARS-CoV-2 pathogenesis. (**A**) Mesenchymal stem cell (MSC) and NK cell-based therapies for the treatment of SARS CoV-2 pathogenesis. Extracellular vesicles (EVs) derived from MSCs or modified exosomes containing antiviral/anti-inflammatory drugs or respective nucleic acid for therapeutic intervention to target cells. These cell-based or cell-derived therapeutic agents could modulate immune cell functions against SARS-CoV-2 infection. (**B**) Cytokine storm development is a central pandemic delinquent in SARS-CoV-2 infection. Certain immunomodulatory agents with excellent safety profiles or current anti-neoplastic interventions (**D**) may be considered for use in combination with antiviral drugs for the treatment of severe or critical COVID-19 cases. (**C**) Importantly, engineered immune cell receptors, including the chimeric antigen receptor (CAR) T-cell, NK cell therapy, offer new therapeutic approaches for SARS-CoV-2 infection. These immune cells collected from recovered patients offer an advantage as the majority of these cells have been primed with a viral antigen before collection and therefore transmit high proliferative efficiency and anti-viral efficacy.

SARS-CoV-2 infection drives a profound cytokine response in the host, comprising a series of mediators that are targeted in immune-mediated inflammatory diseases (IMIDs) (Figure 1). In some patients, a condition of hypercytokinemia also called a cytokine storm with SARS-COV-2 infection develops, which resembles secondary hemophagocytic lymphohistiocytosis, a hyper-inflammatory state triggered by viral infections [35]. Recent reports have shown the level of plasma concentration of pro-inflammatory cytokines, such as TNF-α, IL-1β, IL-6, IL-8, IL-9, IL-10, bFGF, G-CSF, and GM-CSF, as well as chemokines, such as MCP1, IP10, and MIP1α, are elevated in patients with SARS-CoV-2 infection who are either admitted to an intensive care unit (ICU) or non-ICU patients compared to blood from healthy donors. These elevated cytokines levels were associated with lung injury [11,36]. Inflammatory cytokines were significantly elevated in patients who displayed severe clinical conditions and dismal clinical outcomes compared to moderately or mildly infected patients, thus suggesting that extensive changes in cytokines play a pivotal role in COVID-19 pathogenesis [15,37]. The changes in cytokines, immune cell counts, and other associated clinical characteristics from 11 different studies, including a total of 1981 SARS-COV-2-infected patients, have been compared between mild/moderate and severe SARS-COV-2 groups, along with other comorbidities and demographic information of infected patients, which have been provided in Supplementary Tables S1 and S2. As described above, the elevated cytokines level and reduced number of immune cell count were observed.

### 2.4. SARS-COV-2 Severity in Cancer Patients

Besides the development of cytokine storm and exhaustion of immune cells, the clinical outcomes of SARS-CoV-2 infection are also dependent on multiple factors, such as age; clinical morbidities, including metabolic disorders like obesity, diabetes, cardiovascular and liver disease, and other conditions, such as pregnancy and cancer [38,39,40]. Notably, the expression of ACE2 receptors tend to increase with increasing age, and the majority of cancer is diagnosed at near to 60 years of age, marking cancer patients as more prone to SARS-CoV-2 infection. Consequently, that led to adverse clinical outcomes [41]. In addition to changes in the ACE2 receptor, immune functions in cancer patients are compromised. In fact, there is an increase in the expression of immunosuppressive cytokines and markers, such as PD1/PD-L1 on immune cells, which dampened the immune system and augmented the probability of viral infection [42]. Moreover, specific cytokines, such as IL17, secreted by Th17 cells in response to viral infection, have been shown to play a central role in virus pathogenesis through regulating the program cell death, cytokine storm, and lung cancer progression through VEGF (vascular endothelial growth factor) expression stimulation [40,43,44]. Therefore, therapies targeting such cytokines and anti-neoplastic agents might ideally improve the clinical efficacy of SARS-CoV-2-infected cancer patients [45].

### 2.5. Immune Responses of Asymptomatic Patients with SARS-CoV-2 Infection

Immune responses and clinical features of asymptomatic individuals with COVID-19 have not been well defined. These individuals comprise approximately 40–45% of the infected population and can silently spread the virus to others. The absence of symptoms in these infected patients does not mean that they are away from ultimate mortality risk, as they might have viral load equivalent to symptomatic patients. Therefore, more investigations are needed to understand the significant changes in disease symptoms, viral load, proper examination, and immune responses; all this knowledge might be useful to develop directed therapy to prevent the spread of this infection. Compared to asymptomatic people, recovered individuals (virus-negative/antibody-positive) can safely interact with susceptible and infected individuals [46]. Long Q and colleagues studied clinical features and immune responses of 37 asymptomatic individuals (20.8%), identified in a group of total 178 Real time polymerase chain reaction confirmed SARS-COV-2-positive people in the Wanzhou District of China [47]. However, this might not be an accurate assessment of the percentage of asymptomatic infections in the general population, since asymptomatic infections were identified from those who are at high risk of infection and not from a random sample of people. Therefore, the proportion of asymptomatic infections needs to be determined through population screening [48].

According to Long Q et al., the median duration of virus-shedding time in asymptomatic individuals was significantly longer than the symptomatic group. Moreover, the levels of virus-specific immunoglobulin (IgG) and levels of 18 anti-inflammatory cytokines were significantly lower in the asymptomatic group [47]. Previous studies have shown that circulating antibodies against SARS-CoV or MERS-CoV could remain for nearly 3 years or longer [49,50]. Several studies have reported that most SARS-CoV-2 convalescent individuals have detectable neutralizing antibodies, which correlate with the numbers of virus-specific T cells [51,52]. Additional longitudinal serological studies profiling more symptomatic and asymptomatic individuals are urgently needed to determine the duration of antibody-mediated immunity.

## 3. Treatment Options for SARS-CoV-2-Infected Patient

Due to the rapid spread of SARS-CoV-2 infection globally, there is an imperative need for the development of effective therapeutic interventions. Drug development is a time consuming and a long-step process that requires initial safety assessment and further evaluation in multiple phases of clinical trials. The current emphasis of new anti-SARS-CoV-2 therapeutic interventions is to achieve a decrease in viral load, limit hyper-inflammation propagation, reduces disease severity, and improve anti-viral immunity. Table 1 representing current multi-directorial interventions are in clinical trials and depicted in Figure 1.

### 3.1. Convalescent Plasma (CP) Therapy and ACE2 Blocker

CP is a passive immunotherapy technique in which plasma or neutralizing antibodies are purified from the recovered patients and given to the newly infected patients as treatment. CP, when given to SARS-COV-2-infected patients, has shortened the mortality rate, hospital stay, and depletes the viral load within 7 days after treatment [53]. A rapid increase in neutralizing antibodies was observed following transfusion of CP into SARS-CoV-2-infected patients. However, this offers only a short-term immunity to SARS-CoV-2 patients and does not provide long-term protection [54]. Currently, more than 50 clinical trials on CP as a treatment for COVID-19 are on-going—in China (NCT04264858), Sweden, India, United States, and many other countries—on large cohorts, some of which have already reached phase three out of four of a clinical trial, as listed in Table 1 [55,56]. These CPs are usually obtained from donors with confirmed SARS-CoV-2 infection and those who have been symptom free for 14 days. These CPs are a good source of polyclonal neutralizing antibodies, but their outcomes are unpredictable due to serum variability isolated from different patients [57]. There are certain points that need to be considered to improve the efficacy of CP therapy: it ideally necessitates standardizing the antibody titer from donor patients in accordance with the optimal concentration required for effective treatment, dynamic changes in a cytokine storm, and reduction in viral burden. Early intervention of CP before onset or during the initial stage of symptoms will help to improve clinical outcomes compared to those in which CP is given at the time of severe symptomatic condition.

Besides CP, other treatment strategies that have been reported for SARS-CoV-2 are host-directed approaches, such as blocking the ACE2 receptor (lisinopril) or renin-angiotensin-aldosterone system (RAAS) (losartan, telmisartan) of the host cells to prevent viral infection. Using surface spike glycoprotein (S-protein), SARS-CoV-2 enters the host cell by binding to host cells ACE2 receptor. Currently, these angiotensin blockers are in phase three out of four of clinical trials for SARS-CoV-2-infected individuals (NCT04328012, NCT04340557, NCT04394117, NCT04356495, NCT04394117) [58]. A recent study has performed high-throughput molecular docking and screening to investigate an FDA-approved library of pharmacologically active compounds, which target the interphase of SARS-CoV-2 S-protein and ACE2 receptor. A number of compounds were found to bind to the virus binding motifs of the ACE2 receptor (GNF-5, RS504393, TNP, and eptifibatide acetate) and viral S-protein (KT203, BMS195614, KT185, RS504393, and GSK1838705A), respectively [59]. These molecules could also be effective in controlling the rapid spread of SARS-CoV-2 infection in a specific inhibitory manner. It is noteworthy that ACE2 inhibitors are highly recommended medications for patients with cardiovascular diseases, and treatment with ACE2 inhibitors could increase the numbers of ACE2 receptors in their lungs making them high-risk for coronavirus infection [60]. Serine protease inhibitors, such as camostat mesylate, nafamostat mesylate, and gabexate mesylate, act as anti-coagulant for hemodialysis are found to block virus replication [61] and inhibit serine protease TMPRSS2, which is required for SARS-CoV-2 entry into the alveolar cells through viral S-protein priming [62]. Currently, camostat mesylate is in multiple clinical trials in various locations (USA, Denmark, and Israel), which will be given either alone or in combination with salvage therapy for COVID-19-infected patients (NCT04353284, NCT04321096, NCT04374019, NCT04355052).

### 3.2. Targeting Deregulated Signaling in SARS-COV-2-Infected Patients and Treatment Strategies

Interaction of viral coat protein and genomic material to toll-like receptors (TLR3 and TLR7/8) present on innate immune cells triggers the induction of cytokine secretion and activation of multiple antiviral signaling pathways including nuclear factor κB (NF-κB), IFN regulatory factor-3 (IRF3), Janus kinase/signal transducer, and activator of transcription (JAK/STAT) signaling pathways [63]. Identification of such an altered pathway during virus infection will help to unknot the molecular cascade and determine key targetable molecular players. JAK/STAT signaling is a key pathway that transmits cytokine-induced extracellular signaling to intracellular biological effects. IL6 is one of the key cytokines that regulate JAK/STAT pathways and is shown to be up-regulated in COVID-19 patients, thus leading to cytokine storm development [64]. In turn, the JAK/STAT pathway has been shown to regulate IL6 production and leads to aberrant activation of chronic inflammation [65]. It is noteworthy that elevated IL6 levels in circulating blood is an indicator of increased risk of cardiovascular disease, multiple organ dysfunction, and therefore, it is associated with the enhanced detrimental risk to SARS-CoV-2-infected patients. Since IL6 is presented as the driver of cytokine storm in SARS-CoV-2 infection, therapies targeting IL6 such as monoclonal antibodies tocilizumab (Actemra^®^, Genentech), sarilumab (Kevzara^®^, Regeneron/Sanofi), and siltuximab (Sylvant™, EUSA Pharma) is under investigation for COVID-19 treatment either alone or in combination in multiple clinical trials (more than 50 to date), and 11 of them are already in phase three out of four of a clinical trial (NCT04412772, NCT04377750, NCT04356937, NCT04372186). Pharmacology, pharmacokinetics, clinical efficacy, and the safety of tocilizumab in rheumatoid arthritis (RA) have been well-established previously [66]. Though tocilizumab is effective in reducing the cytokine storm and provided beneficial clinical outcomes in combination with other therapies, noticeable cases of refractory to tocilizumab treatment are presented, and therefore, alternative biological agents to overcome inflammatory cascade are necessary [67]. In scenario tocilizumab refractory cases, blocking of the JAK/STAT pathway, which is the primary regulator of IL6 signaling with specific inhibitors (baricitinib, fedratinib, tofacitinib, peficitinib, and ruxolitinib) could provide an alternative strategy to attenuate the host inflammatory response associated with SARS-CoV-2 infection [67,68]. To date, there are several clinical trials driving safety and efficacy of JAK/STAT inhibitors: clinicaltrials.gov.

NF-kB is a critical regulator of both innate and adaptive immune responses as this signaling activates several cytokines’ gene expression and anti-apoptotic proteins. Therefore, it is required for optimal immune responses [69]. Activation of the NF-kB pathway is often associated with inflammatory disease, B-cell malignancies, and rheumatoid arthritis [70]. Notably, the upregulation of IL6 and TNFα in SARS-CoV spike protein-induced murine macrophage was shown to be regulated via activation of NF-KB signaling [71]. As a therapeutic intervention, pharmacological inhibition of the NFkB pathway provided protective responses from SARS-CoV infection, suggesting that NF-kB inhibition might be an effective strategy to block COVID-19 pathogenesis [72]. However, this is also limited by an escape mechanism mediated by viruses that manipulate NF-kB signaling through their multifunctional viral proteins [73]. Therefore, a potential strategy to target NF-kB signaling in case of viral infection need to be considered, such as those that target NF-kB downstream effector molecules; TNFα was found to be associated to SARS-CoV infection through TNFα-converting enzyme (TACE)-dependent shedding of the ACE2 receptor during virus entry [74]. Monoclonal antibodies targeting TNFα (infliximab, adalimumab) are currently in clinical trials for SARS-CoV-2-infected patients (NCT04425538, NCT04344249).

### 3.3. Regenerative Medicine in COVID-19 Treatment

Novel coronavirus infection has gained global attention due to its rapid transmission and high mortality rate. Therefore, to achieve an effective therapeutic regimen, massive research in multiple directions and clinical trials are on-going throughout the world. Regenerative medicine offers new therapeutic tools and related products, such as stem cell therapy, CAR T cell therapy, natural killer (NK) cell therapy, exosomes, and other tissue products. Notably, among these, mesenchymal stem cell (MSC)-based therapies are now being considered in multiple clinical trials (Table 1).

#### 3.3.1. Stem Cell-Based Therapeutic Interventions

Stem cell-based therapies have become a promising therapeutic approach, particularly for a disease that is challenging to treat. Notably, stem cells including pluripotent or multipotent cells are found to be resistant to viral infection and associated with intrinsically expressed interferon-gamma-stimulated genes [75]. As direct evidence, human hematopoietic cells are shown to be non-permissive to Myxoma virus infection [76]. There are several stem cell-based clinical trials (37 trials to date) to treat COVID-19 that have been started in several countries, including the USA, China, Spain, and Brazil, registered on ClinicalTrials.gov [77]. The details of these 33 clinical trials have been described by Tsuchiya et al. [77]. Among the various population of stem cell types, MSCs are representing the most promising candidate for the treatment of SARS-CoV-2 infections due to their low invasive acquisition procedure, high proliferation rate, ease of expanding, maintaining and cryopreserving, and they can be isolated from several tissues (peripheral blood, cord blood, dental pulp, adipose tissue, bone marrow, fetal liver, etc.). MSCs have immunomodulatory, anti-fibrotic, anti-oxidant, and anti-inflammatory properties and, therefore, may inhibit the cytokine storm, which is a foremost clinical concern of SARS-CoV-2 infection [78]. A clinical trial of seven COVID-19 patients has proven the therapeutic efficiency of intravenously transmitted MSCs in improving immunological functional and clinical outcomes [79]. A case report of SARS-CoV-2 patients presented improvement in immunomodulation (increase in T cell subsets count and decrease in inflammatory cytokines levels) after treatment with human umbilical cord Wharton’s jelly-derived MSCs isolated from a healthy donor [80]. It is worthwhile to note that the clinical efficacy of MSCs depends on generally how the MSCs have been prepared, the source of preparation, expansion protocol, and pre-treatment with pharmacological factors, which can further improve the therapeutic effects of MSCs [81].

#### 3.3.2. Extracellular Vesicles (EVs) for COVID-19 Treatment

EVs are nano-sized lipid membrane-enclosed cell-secreted particles, which are utilized for cellular communications by transferring information in the form of protein and nucleic acid. EVs are present in all body fluids and secreted by all cell types. Therefore, these EVs may contribute to the spread of SARS-CoV-2, as they can transfer the ACE2 receptor from one cell to another or different cell types [82]. EVs can be used as a delivery vehicle for chemotherapeutic agents, siRNA, and for transfer of critical cellular information in a lipid-bound protective format to distant recipient target cells. As a therapeutic purpose, the delivery of human recombinant soluble ACE2 (hrsACE2) through EVs for ARDS has been shown to be effective in reducing cytokine storm (NCT01597635) [83]. Therefore, it is speculated that the delivery of EVs encapsulated ACE2 receptor would likely increase the efficiency in neutralizing SARS-CoV-2 infection [84].

Bone marrow or MSC-derived EVs have anti-inflammatory, immunomodulatory, pro-angiogenic, and anti-fibrotic properties and, therefore, can act as a therapeutic regimen for SARS-CoV-2 and have shown promising results in multiple malignancies [85,86]. Importantly, MSC-derived EVs have been identified to attenuate influenza virus-induced acute lung injury in pigs by preventing virus shedding, replication, and virus-induced production of pro-inflammatory cytokines [87]. In line with this, a clinical trial was carried out in which SARS-CoV-2-infected patients (n = 24) were given exosomes (ExoFlo™) intravenously, which were derived from allogeneic bone marrow MSCs. Out of 24 patients, 17 (71%) were completely recovered with a significant increase in neutrophil, CD4+, CD8+ T cell count (46%), and decrease in C-reactive protein levels in the blood indicating MSC-EVs can be a promising therapeutic candidate for severe SARS-CoV-2 infection with the capacity to restore oxygenation and downregulate cytokine storm [88]. Another clinical trial that is underway has intended to deliver MSC-derived EVs through inhalation to SARS-CoV-2-infected patients (NCT04276987). It is important to note that not only EVs derived from MSCs can be utilized for therapeutic purposes, and certain antiviral, anti-inflammatory, or other drugs can also be loaded into EVs for disease therapy. Therefore, repurposing these drugs with a proper formulation into EVs may improve the safety and efficacy of COVID-19 treatment.

#### 3.3.3. NK Cell-Based Therapy

NK cells are major immune cells and require the development of both innate and adaptive immune response through their cytotoxic activity against virus-infected cells and cancer cells. SARS-CoV-2-infected patients with severe disease have reduced NK cell count, and these exhausted NK cells showed an increased expression of suppressing receptor CD94/NK group-2 member A (NKG2A) in contrast to NKG2D (an activating receptor of NK cells) [89]. A previous study has demonstrated that treatment with soluble IL15 can functionally enhance circulating NK cell count and induces its antibody-dependent cellular cytotoxicity (ADCC) activity [90]. Using all this knowledge, a CAR NK has been designed (NKG2D-ACE2-CAR-NK) and is currently in phase one out of two of a clinical trial involving 90 SARS-CoV-2-infected participants (NCT04324996), with main objectives being to check efficacy, safety, and tolerability of NK CAR products. NKG2D-ACE2-CAR-NK cells have been derived from cord blood cells and designed to secrete IL15 super-agonist, which could increase the sustainability and activity of infused NK cells, and secrete GM-CSF neutralizing scFv, which could abrogate cytokine storm and associated neurotoxicity. Another phase one out of two clinical trial (NCT04365101) involving 72 COVID-19 patients has been recruited to determine the safety and efficacy of CYNK-001, immunotherapy containing NK cells derived from human placental CD34+ cells and culture-expanded. Other NK cell-dependent clinical trials underway have been demonstrated in Table 1.

#### 3.3.4. Chimeric Antigen Receptor (CAR) T-Cell Therapy

CAR T therapy represents an adoptive form of T cell-based therapy, where T cells are reprogrammed to express synthetic receptor directed against the specific antigen. CAR T therapy has shown significant promising improvement, specifically in B-cell malignancies [70] and also in solid tumors [91]. Through early encouraging results, recent studies have demonstrated the success of CAR T therapy against viral infection, including chronic hepatitis B-virus (HBV) infection and HBV related hepatocellular carcinoma [92,93]. Therefore, scientists are now investigating the prospects of CAR T therapy against COVID-19 disease. Janice et al. have engineered CD8+ lymphocytes from healthy individuals into SARS-CoV-specific CD8+ T cells by using CD8+ memory T cell receptors isolated from previously recovered SARS-CoV-infected individuals. These engineered SARS-CoV CD8+ T cells displayed functional profile and avidity comparable to that of natural SARS-specific memory CD8+ T cells from recovered individuals [94] and, therefore, providing a rationale to design or engineer SARS-CoV-2-specific adaptive therapies. Notably, CD8+ T cells are essential for protection against virus and a high level of virus-specific CD8+ T cells is correlated to decrease viral load and favorable clinical outcomes. However, adoptive T cell transfer therapy also has multiple obstacles: the first caveats are the genetic restriction for allogeneic T cell transfer from an unrelated individual, second is T cell exhaustion or failure to proliferate after infusion, the third is the creation of massive side-effects such as cytokine storm, and the forth obstacle is excessive proliferation of infused T cells that could lead to the enhanced killing of virus-infected cells and enormous tissue injury. Therefore, it is important to consider these obstacles before designing a novel CAR product for adoptive therapy. In line with this, HLA-E-restricted unconventional CD8 T cell therapy against SARS-CoV-2 may offer several advantages to improve the ability to kill virus-infected cells, limiting the extent of inflammatory responses and tissue damage [95]. HLA-E is a non-classical major histocompatibility complex Class-I molecule characterized by limited polymorphism and, therefore, will have limited alloreactivity responses. Recently, investigators from Singapore have started a clinical trial to determine the efficacy of adoptive cell therapy on severely infected SARS-CoV-2 patients (https://www.clinicaltrials.gov/ct2/show/NCT04351659). Though CAR T cell therapy is found to be effective against B-cell lymphoma, only very few CAR T cell-based studies are now in clinical trials for SARS-CoV-2 infection, which suggests that there are numerous obstacles that require clinical optimizations for successful generation of anti-SARS-CoV-2 CAR T therapy. The workflow of CAR therapy generation has been described in Figure 1.

### 3.4. Antineoplastic Therapy for SARS-CoV-2-Infected Patients

It is noticeable that cancer patients due to compromised immune responses have a worse clinical outcome and four times higher risk of hospitalization compared to other subjects when infected with SARS-COV-2. Therefore, anti-cancer agents should be carefully given to these populations. Importantly, anti-cancer therapy modulates the immune system and thereby might be beneficial to at least SARS-CoV-infected healthy individuals (Table 2). As the development of new vaccines usually takes several years, existing drugs including FDA-approved anti-cancer agents are now in clinical trials for COVID-19 treatment. Corticosteroids such as dexamethasone and prednisolone, which are utilized for the treatment of lymphoma and leukemia’s are now in phase ¾ clinical trials as mono or combination therapy with other anti-viral agents. Immunomodulatory drugs, such as lenalidomide (Revlimid) and thalidomide generally used for multiple myeloma, lymphoma, graft-versus-host disease, and myelodysplastic syndromes, work through multiple mechanisms such as decreasing TNF-α production and stimulating T cells’ function [96]. A case report of 45-year-old women with SARS-CoV-2 infection who were treated with thalidomide in combination with low-dose short-term glucocorticoid showed dramatic inhibition of cytokine surge, with an absolute increase in lymphocyte counts and achieved relieve from digestive imbalance [97]. Furthermore, clinical trials with these immunomodulators for the treatment of COVID-19 are underway. Selinexor (Karyopharm, Newton, MA, USA) is a nuclear export inhibitor that binds to the exportin 1 and prevents the translocation of several anti-apoptotic proteins (from the nucleus to cytoplasm) involved in cancer progression [98]. This nuclear export inhibitor could retain SAR-CoV2 essential viral proteins into the host nucleus, thus suppressing its virulence and pathogenesis [99]. The FDA has approved Selinexor for relapsed/refractory multiple myeloma in combination with dexamethasone, and now this drug is in a clinical trial for SARS-CoV-2-infected patients [98].

Cytotoxic chemotherapy, such as DNA damaging agents targeting topoisomerase-2A etoposide, is considered as primary chemotherapy for the treatment of several cancers including leukemia, neuroblastoma testicular, lung cancer, lymphoma, and ovarian cancer [100]. Notably, a clinical trial for a small cohort of SARS-CoV-2-infected patients, salvage treatment along with etoposide in adjunction to immunosuppressants resulted in an overall favorable outcome (NCT04356690) [101]. Therefore, an etoposide-based treatment strategy might be clinically acceptable as another salvage approach for severely SARS-CoV-2-infected individuals [102]. The chemotherapeutic drug methotrexate in combination with salvage therapy has proven to be effective in immnunostabilization for SARS-CoV-2-infected individuals. In fact, high doses of methotrexate along with leucovorin (drug decrease toxic effect of methotrexate) can rescue a SARS-COV-2-triggered panic attack [103]. Vascular endothelial growth factor (VEGF) is the most potent vascular permeability inducer and has been found to be unregulated in SARS-CoV-2-infected patients and identified as the most important indicator of the severity of SARS-CoV-2 infection [104,105]. Bevacizumab (Genentech, San Francisco, CA, USA) is a humanized monoclonal antibody that inhibits VEGF-A and is currently in clinical trials in China and Italy (Table 2).

Small molecular kinase inhibitors are widely used in cancer therapeutics; they inhibit respective specific tyrosine or serine-threonine kinase and, thereby, block oncogenic signaling. One such widely used FDA-approved small molecular irreversible inhibitor of Bruton tyrosine kinase (BTK) “ibrutinib” has extensively shown its clinical efficacy in B-cell lymphoma, such as mantle cell lymphoma (MCL), chronic lymphocytic leukemia (CLL), and diffuse large B-cell lymphoma (DLBCLs) [70]. Ibrutinib inhibits BTK-suppressed activation of NF-kB signaling and thus prevents expression of multiple cytokines gene expression [70]. Recently, Treon et al. have elucidated the efficacy of ibrutinib in which five COVID-19 patients were given ibrutinib at the dose recommended for the treatment of lymphoma (420 mg/day). Ibrutinib treatment reduced the expression of pro-inflammatory cytokines; the patients did not experience dyspnea and did not require hospitalization [106]. Another group investigated the effects of acalabrutinib (2nd generation of BTK inhibitor) on disease progression of SARS-CoV-2-infected patients. With a cohort of 19 severe COVID-19 patients, treatment with acalabrutinib improved oxygenation balance in the majority of patients and significantly reduced C-reactive protein and IL6 levels [107]. Though a small cohort was investigated for ibrutinib efficacy, it surely undoubtedly offers interesting possibilities as combinatorial treatment for SARS-CoV-2 treatment in a large cohort. Alemtuzumab is a monoclonal antibody that acts as an immunosuppressive drug, which binds to CD52, a protein present on mature lymphocytes and is in clinical treatment for CLL and multiple sclerosis [108]. A recent study for the management of clinical multiple sclerosis along with SARS-CoV-2 infection represented alemtuzumab treatment could attenuate COVID-19 associated symptoms and induce positive changes in the immune functions against SARS-CoV-2 infection [109]. Further clinical investigations are needed to conclude the potentials of alemtuzumab as a therapeutic regimen for COVID-19. PI3K/Akt signaling has been widely studied as one of the most critical intracellular pathways considered to be critically upregulated in chemo-resistant cancer patients [70,110]. A number of pan-PI3K and PI3K isoform-specific inhibitors have been available, and some of them are in clinical trials for multiple cancer types [110]. Importantly, inhibition of the PI3K/Akt/mTOR pathway has shown a significant reduction in MERS-CoV replication in vitro previously [111,112]. Therefore, targeting the PI3K pathway may represent a novel therapeutic intervention for SARS-CoV-2 infection. Duvelisib (PI3K inhibitor) is currently under investigation in a clinical trial for SARS-CoV-2-infected patients (NCT04372602).

Immune check point inhibitors, such as pembrolizumab, nivolumab treatment, inhibits programmed death receptor-1 (PD1) and its ligand PD-L1 axis, which restores anti-tumor immunity, transforms the therapeutic outcomes and, therefore, is utilized as a therapeutic regimen in multiple cancers [113]. A case study is presented by Emre et al. of an old lady who had metastatic malignant melanoma and was infected with SARS-CoV-2. Treatment with nivolumab provided her with an excellent clinical condition even though she had multiple co-morbidities [114]. Similar observations reported in another case study with pembrolizumab representing favorable clinical outcomes might be associated with the PD1/PD-L1 blockade [115]. However, further clinical trials with these checkpoint inhibitors are needed to be recruited to achieve a defined conclusive efficacy.

### 3.5. Vaccine Development for COVID-19

Like other vaccines, the development of an effective vaccine against SARS-CoV-2 that can prevent the spread of infection, prevent new infections, and can provide immunity to people will be the most economical and long-term therapeutic strategy for COVID-19 prevention. Based on the current stressful situation and availability of advanced technologies has provided a platform for accelerated development of the new regimen; some of these are currently in clinical trials (Table 3). These vaccines are derived from a diverse platform, such as a DNA-based, mRNA-based, adenovirus-based, and inactivated virus-based vaccine. DNA and mRNA-based vaccines offer a great advantage; they can encode for antigenic proteins, feasibility for stress-free antigen manipulation, rapid production, and the availability of multiple delivery technologies, such as electroporation and lipid nanoparticle-based and adenovirus-based vectors [116]. However, a DNA vaccine also imparts certain shortcomings if we compared it to mRNA-based vaccines, since it can potentially integrate into the human genome, have reduced immunogenicity, instability, and induction of anti-vector immunity [117]. Numbers of clinical trials are on-going for mRNA and DNA-based vaccines and are going to be in phase 3 clinical trials for mainly RNA-based vaccines (mRNA-1273) [118].

Adenoviral vector-based vaccines provides another platform to correct malignancies, including infectious diseases and cancers that are now in clinical trials [119]. There are certain advantages that adenovirus-based vectors have over the viral vectors: a broad range of tissue tropism, ease of genetic manipulation, inherent adjuvant properties, induces robust transgene-specific immune response, non-replicative in the host, and can be grown in high titer in culture. There are a couple of drawbacks associated with adenovirus-based vaccines: immunodominance against the vector gene over transgene, pre-existing immunity, sequester of the vector in liver and spleen, and inflammatory response. So far, several adenoviral-vector-based vaccines have been developed against SARS-CoV-2 infection, and those will be tested in healthy volunteers (listed in Table 3). The first generation of COVID-19-specific adenovirus-based vaccine (Ad5) presented the induction of neutralizing antibodies [120]. Second generation and other adenovirus vaccines from China, Canada, and Russia for COVID-19 are underway in clinical trials (Table 3).

Inactivated vaccines are made from pathogens that have been killed by physical or chemical treatment, and they do not have the replicative ability. Compared to live attenuated vaccines, the inactivated vaccine can be easy to produce, more easily stored, and are incapable of causing disease. However, the inactivated vaccine induces weaker immune responses compared to live attenuated vaccines. An inactivated vaccine for COVID-19 has been developed by China, and this is in phase one out of two of clinical trials (NCT04412538, NCT04352608). COVAXIN^TM^ is another inactivated vaccine for COVID-19 developed in India by Bharat Biotech in collaboration with the Indian Council of Medical Research (ICMR) and National Institute of Virology (NIV). This indigenous inactivated vaccine received Drug Controller General of India approval for phase I and II Human Clinical Trials, and the trials will commence across India from July 2020.

Dendritic cells (DCs) are considered as the most effective antigen-presenting cells (APC), being almost 10–100 times more potent than the other APCs (monocytes and B-cells), capable of priming antigen to naïve T-cells and induce T-cell proliferation [121]. The DCs can be genetically engineered by RNA interference (RNAi), CRISPR, and other gene-editing techniques, to modify their chimeric receptor, cytokine secretion, specific antigen presentation with ultimate effects onto improving their anti-tumor efficacy and immunity [122].

## 4. Conclusions

SARS CoV-2 infection as of writing has still been marked as a pandemic scenario throughout the world, with the United States, Brazil, and India being the top three leading countries in the COVID-19 pandemic. Even though scientists and medical experts are working round the clock to develop an effective treatment. Multiple therapeutic strategies so far have been attempted in the past six months to prevent the spread of SARS-CoV-2 infection and provide overall benefits to infected people. The ultimate goal of our government, researchers, and clinicians are to provide an efficacious vaccine so that our new generation will be safe in the future from this deadly infection. So far, plasma and stem cell-based therapies and vaccines are pharmaceutical interventions that are being actively explored. Several kinase inhibitors or drugs re-considered for COVID-19 therapy are in use for cancer therapy. The immunological events induced by SARS-COV-2 infection and cancer relatively similar and, therefore, the above-mentioned anticancer drugs, chemokine/cytokines inhibitors, are also more extensively recruited in clinical trials. Although lessons from the previous SARS and MERS epidemics can be drawn, there is still much to do to conclude whether the SARS-CoV-2 behaves in the same way as its predecessors or if peculiar specificities characterize it. We hope that these anticancer drugs or novel vaccines may pave the way for a more comprehensive clinical experimentation on repurposing with FDA-recommended antiviral drug combinations for the treatment of COVID-19, a line of research sustained by limited funds but of prime importance to face this new worldwide challenge.

## Figures and Tables

**Table 1:** Selected clinical studies using stem cells, adoptive cells, and extracellular vesicle-based treatment approaches for SARS-CoV-2 infection.

Trial No	Clinical Stage	COVID-19 Patient (n)	Interventions	Primary Endpoint (day)	Brief Description of Study	Location
NK Cell-Based Therapy						
NCT04324996	Phase (I/II)	90	NKG2D CAR-NK cells, NKG2D-ACE2 CAR-NK cells (10^8^ cells/kg, IV)	CR, safety and tolerability (day 28)	NK cells from umbilical cord blood and engineered genetically, secreting IL15 and GM-CSF.	China
NCT04375176	Recruiting	150	Functional analysis of monocytes and NK cells	Immune cells activity (6 months)	Phenotypic and functional analysis of monocytes and NK cells	Italy
NCT04280224	Phase (I)	30	NK cells (0.1–2 × 10^8^ cells/kg, twice per week)	CR, safety and tolerability (day 28)	NK cells in combination with standard therapy	China
NCT04365101	Phase (I/II)	86	CYNK-001 allogeneic NK cells (days 1, 4, and 7)	AE and clinical improvement (day 28)	CD56+/CD3- NK cells derived from human placental CD34+ cells and culture-expanded	USA (multiple locations)
NCT04363346	Phase (I)	12	FT516 (9 × 10^7^ cells day-1, 3 × 10^8^ cells day-4, 9 × 10^8^ cells day-7).	MTD of FT516 using 3 dose-escalation strategies, Dose Limiting Toxicity Events (day 36)	NK cell product derived from an iPSC transduced with ADAM17 non-cleavable CD16	USA
NCT04344548	Phase (I/II)	10	Three doses of allogeneic NK cell transfer	AE and safety	Safety and immunogenicity of allogeneic NK cells from PBMCs of healthy donors in patients infected with COVID-19 collected by apheresis	Colombia
Mesenchymal Stem Cell-Based Therapy in Phase ¾ of Clinical Trial						
NCT04444271	Phase (II)	20	MSCs, 2 × 10^6^ cells/kg, day 1 and 7	Overall survival	Efficacy of MSCs as an add-on therapy to standard supportive treatment	Pakistan
NCT04416139	Phase (II)	10	MSCs 1 million/kg single dose, IV	CR, safety and tolerability	Efficacy of MSCs	Mexico
NCT04366063	Phase (II/III)	60	Two doses MSCs 100 × 10^6^, IV+CT.	AE and safety, 28 days	MSCs for ARDS	Iran
NCT04336254	Phase (I/II)	20	3 × 10^7^ human dental pulp stem cells, day 1,4,7	CR, safety and tolerability	Human dental pulp MSCs	China
NCT04366323	Phase (I/II)	26	Two doses, 80 million MSCs	AE and safety, 28 day	Allogeneic adult MSCs of expanded adipose tissue	Spain
NCT04366271	Phase (II)	106	1 infusion of undifferentiated allogeneic MSCs	AE and safety, 28 days	MSCs from umbilical cord tissue	Spain
NCT04390139	Phase (I/II)	30	2 infusions of Wharton-Jelly MSCs, 1 × 10^6^ cells/kg.dose	AE and safety, 28 days	Drug: XCEL-UMC-BETA	Spain
NCT04333368	Phase (I/II)	40	1 million/kg, day 1,3,5	CR, safety and tolerability	Umbilical Cord-derived MSCs	France
Convalescent Plasma in Phase ¾ of Clinical Trial						
NCT04348656	Phase (III) or Phase (II/III)	1200	200–500 mL ABO compatible CP plasma or two doses of 250 mL CP	Recovery or decrease mortality in hospital	CONCOR-1 trial for efficacy of transfusion of COVID-19 CP	The multicenter USA and Canada
NCT04362176		500			Passive Immunity Trial of Nashville II	
NCT04376034		240			CP treatment in pediatric and adults	
NCT04361253		220			Infusion of high-titer COVID-19 CP	
NCT04425915		400				India
NCT04342182		426			CONCOVID Study	Netherlands
NCT04374526		182			LIFESAVER	Italy
NCT04385043		400			COV2-CP	
NCT04345289		1500			Six parallel treatment arms of CP, sarilumab, hydroxychloroquine, baricitinib, IV and SC	Denmark
Extracellular Vesicle as COVID-19 Therapy						
NCT04276987	Phase (I)	30	5 times aerosol inhalation of MSCs-derived Exo (2 × 10^8^ particles)	AE, safety and tolerability (day 28)	Exo derived from allogeneic adipose MSCs	China
NCT04384445	Phase (I/II)	20	Organicell Flow, 2–5 × 10^11^ particle/mL, IV.	AE, safety and tolerability (day 60)	Organicell Flow derived from human amniotic fluid contain chemokines, cytokines and EVs	USA
NCT04389385	Phase (I)	60	5 times aerosol inhalation of CSTC-Exo (2 × 10^8^ particles)	AE, safety and tolerability (day 28)	CSTC-Exo, in vitro expanded and cultured with virus peptide and GFs.	Turkey
Adoptive Cell Therapy (Chimeric Antigen Receptor CAR Therapy)						
NCT04351659	NA	8	Donors who had tested positive for SARS-CoV-2 in the past and have recovered are suitable for blood donation.	The success rate in production of SARS-CoV-2 specific T cells from a convalescent donor	Development of treatment protocol for adoptive T-cell therapy	Singapore

Abbreviations: IV, intravenous infusion; CR, clinical response; AE, adverse events; MTD, maximum tolerated dose; CP, convalescent plasma; NA, not applicable; SC, subcutaneous; EVs, extracellular vesicles; MSCs, mesenchymal stem cells; GFs growth factors; Exo, exosomes; CSTC, COVID-19-specific T cell-derived; PBMC, peripheral blood mononuclear cells; CT, conventional treatment; ARDS, acute respiratory distress syndrome.

**Table 2:** Selected clinical trials on-going using anti-neoplastic agents for SARS-CoV-2 infection.

Drug Class	Active Agent	Targetable Action	Cancer Treatment	Trial Number	Size	Clinical Phase
Corticosteroid	Dexamethasone	Anti-inflammatory Immunosuppressive	Lymphomas, leukemias	NCT04325061	200	Phase 4
				NCT04347980	122	Phase 3
	Methylprednisolone			NCT04341038	84	Phase 3
				NCT04438980	72	Phase 3
				NCT04244591	80	Phase 3
				NCT04263402	100	Phase 4
Immunomodulators	Lenalidomide	Antiangiogenic, TNF-α signaling modulation	Multiple myeloma, myelodysplastic syndromes	NCT04361643	120	Phase 4
	Thalidomide			NCT04273529	100	Phase 2
				NCT04273581	40	Phase 2
Nuclear export inhibitor	Selinexor	Binds to exportin 1	Relapsed/refractory multiple myeloma	NCT04355676	80	Phase 2
				NCT04349098	230	Phase 2
Chemotherapy	Methotrexate	Antimetabolite for antifolate type	Multiple cancers including breast, lymphoma, epidermoid, small-cell lung cancer, head and neck, squamous cell lung cancer.	NCT04352465	42	Phase ½
	Etoposide	Inhibits topoisomerase-II		NCT04356690	64	Phase 2
Anti-vascular endothelial growth factor	Bevacizumab	The humanized monoclonal antibody that blocks angiogenesis by inhibiting VEGF-A	Number of types of cancers and a specific eye disease	NCT04305106	140	NA
				NCT04344782	130	Phase 2
				NCT04275414	20	Phase 2/3
Immune check-point inhibitors	Pembrolizumab	Block PD1 receptor	Multiple cancer types	NCT04335305	24	Phase 2
	Nivolumab			NCT04413838	120	Phase 2
				NCT04343144	92	Phase 2
				NCT04356508	15	Phase 2
Kinase inhibitors	Ibrutinib	Irreversible covalent BTK inhibitor	B-cell lymphoma, including CLL, MCL, ABC-DLBCLs	NCT04375397	46	Phase 2
				NCT04439006	72	Phase 2
	Acalabrutinib			NCT04380688	60	Phase 2
				NCT04346199	140	Phase 2
	Duvelisib	inhibitor of PI3K	CLL, SLL	NCT04372602	25	Phase 2
	Imatinib	Inhibits bcr-abl tyrosine kinase	CML, ALL	NCT04394416	204	Phase 3
				NCT04422678	30	Phase 3
				NCT04346147	165	Phase 2

Abbreviations: PD1, program cell death-1; TNF, tumor necrosis factor; VEGF, vascular endothelial growth factor; BTK, Bruton tyrosine kinase; CLL, chronic lymphocytic leukemia; MCL, mantle cell lymphoma; ABC-DLBCLs, activated B-cell like diffuse large B-cell lymphoma; SLL, small lymphocytic leukemia; PI3K, phosphatidylinositol 3-kinase; CML, chronic myelogenous leukemia; ALL, acute lymphocytic leukemia. Note: “Size” represented the number of participants enrolled in the given clinical trial.

**Table 3:** Selected list of clinical trials on curated vaccine production platform and respective targets for COVID-19.

Company/Sponsor	Vaccine	Trial Number	Size	Clinical Stage	Brief Description	Intervention	Location
DNA-Based Vaccine for COVID-19							
Genexine, Inc.	GX-19	NCT04445389	190	Phase ½	For safety, tolerability, and immunogenicity in HV	Expressing SARS-CoV-2 S-protein antigen, IM via EP	Korea
Inovio Pharma.	INO-4800	NCT04336410	120	Phase 1	For safety, tolerability, and immunological profile in HV	A Prophylactic Vaccine Against SARS-CoV-2, ID then EP	USA
Symvivo Corp.	bacTRL-Spike	NCT04334980	112	Phase 1	For safety, tolerability, and immunity in HV	Bacterial medium with live Bifidobacterium longum, with plasmid expressing SARS-CoV-2 spike protein, 1–10 billion CFU	USA, Canada
Zydus Cadila	ZyCoV-D	-	-	Phase ½	For safety, tolerability		India
mRNA-Based Vaccine for COVID-19							
CureVac AG	CVnCoV	NCT04449276	168	Phase 1	Reactogenicity, Immunogenicity in HV	Participants will receive an IM injection in deltoid area	Germany
NIAID,	mRNA-1273	NCT04283461	155	Phase 1	Safety Immunogenicity in HV	LNP dispersion containing mRNA for spike protein of SARS-CoV-2, IM, 10–250 microgram	USA
ModernaTX,Inc.	mRNA-1273	NCT04405076	600	Phase 2	Dose-confirmation study in HV	mRNA-1273: 50 microgram, IM	USA
Biontech SE	BNT162	NCT04368728	7600	Phase ½	Safety, tolerability, immunogenicity in HV	Anti-viral RNA vaccine for active immunization against COVID-19, IM various doses	USA
Adenovirus-Based Vaccine for COVID-19							
PLA of China	Ad5-nCoV	NCT04341389	508	Phase 2	Immunogenicity and safety in HV	Encodes for a full-length spike (S) protein of SARS-CoV-2, IM, 0.5–1 × 10^11^ VP	China
CanSino Biologics	Ad5-nCoV	NCT04313127	108	Phase 1	Dose-escalating, in HV	IM, 0.5–1.5 × 10^11^ VP	China
CanSino Biologics	Ad5-nCoV	NCT04398147	696	Phase ½	Safety, tolerability, and immunogenicity, in HV	Single dose, 5–10 × 10^10^ VP, IM	Canada
Gamaleya Research Institute	Gam-COVID-Vac Lyo	NCT04437875 NCT04436471	38	Phase 1–2 *	Safety, tolerability, and immunogenicity in HV	rAd26, type 26 adenovirus, containing the SARS-CoV-2 S protein gene, IM	Russia
Altimmune, Inc.	NasoVAX	NCT04442230	96	Phase 2	Safety and effectiveness in early COVID-19-infected people	Replication-deficient adenovirus vectors in suspension	USA
Inactivated SARS-CoV-2							
Chinese Academy of Medical Sciences	Unname	NCT04412538	942	Phase ½	Safety and immunogenicity in HV	SARS-CoV-2 inactivated vaccine, two doses at 0, 28 day, 50–150 U/0.5 mL	China
Sinovac R & D Ltd.	Unname	NCT04352608	744	Phase ½	Safety and immunogenicity in HV	Two doses at day 0, 28, 600–1200 SU/0.5 mL	China
Bharat Biotech	COVAXIN	-	-	Phase ½	Safety and immunogenicity	-	India
Dendritic cell-based vaccine for COVID-19							
Shenzhen Geno-Immune Medical Institute	LV-SMENP-DC	NCT04276896	100	Phase ½	Covid-19 minigenes engineered using lentiviral vector system (NHP/TYF) to modify DCs	5 × 10^6^ LV-DC vaccine and 1 × 10^8^ CTLs via sub-cutaneous injections and IV infusions respectively	China

Abbreviations: HV, healthy volunteers; IM, intramuscular; ID, intradermal; EP, electroporation; CFU, colony-forming unit; VP, viral particles; NIAID, National Institute of Allergy and Infectious Diseases; LNP, lipid nanoparticles; DCs, dendritic cells; LV-DC, lentiviral modified dendritic cells expressing viral proteins and immune modulatory genes; CTLs, cytotoxic T-cell lymphocytes. Note: “Size” represented the number of participants enrolled in the given clinical trial. * Gam COVID-Vac Lyo is licensed and approved by the Russian Government after phase 1–2 combined clinical trials for production. While claims were made to combine production along with phase 3 trials [123].

## Supplementary Materials

The following are available online at https://www.mdpi.com/2079-7737/9/9/243/s1, Table S1: Cytokines and immune cells comparison between mild and severe SARS-CoV-2-infected patients. Table S2: Clinical symptoms and other comorbidities.
